# Comparable Efficacy of Lopinavir/Ritonavir and Remdesivir in Reducing Viral Load and Shedding Duration in Patients with COVID-19

**DOI:** 10.3390/microorganisms12081696

**Published:** 2024-08-16

**Authors:** Choon-Mee Kim, Jae Keun Chung, Sadia Tamanna, Mi-Seon Bang, Misbah Tariq, You Mi Lee, Jun-Won Seo, Da Young Kim, Na Ra Yun, Jinjong Seo, Yuri Kim, Min Ji Kim, Dong-Min Kim, Nam-Hyuk Cho

**Affiliations:** 1 Premedical Science, Chosun University College of Medicine, Gwangju 61452, Republic of Korea; 2 Health and Environment Research Institute of Gwangju, Gwangju 61954, Republic of Korea; jkchung62@naver.com (J.K.C.);; 3 Department of Internal Medicine, Chosun University College of Medicine, Gwangju 61452, Republic of Koreaktandms@gmail.com (M.-S.B.);; 4 Department of Microbiology and Immunology, Seoul National University College of Medicine, 103, Daehak-ro, Jongno-gu, Seoul 03080, Republic of Korea; 5 Institute of Endemic Disease, Seoul National University Medical Research, Seoul 03080, Republic of Korea; 6 Seoul National University Bundang Hospital, Seongnam 13620, Republic of Korea

**Keywords:** COVID-19, SARS-CoV-2, lopinavir/ritonavir, remdesivir, real-time reverse transcription-polymerase chain reaction, viral shedding

## Abstract

The spread of COVID-19 has significantly increased research on antiviral drugs and measures such as case isolation and contact tracing. This study compared the effects of lopinavir/ritonavir and remdesivir on COVID-19 patients with a control group receiving no antiviral drugs. Patients confirmed to have a SARS-CoV-2 infection via real-time RT-PCR were divided into three groups: lopinavir/ritonavir, remdesivir, and control. We assessed the efficacy of these drugs in reducing viral load and viral shedding duration using real-time RT-PCR and Vero E6 cell cultures. Lopinavir/ritonavir led to no detectable infectious SARS-CoV-2, with a median viral clearance time of one day, whereas one remdesivir-treated case remained culture-positive until day 12. Lopinavir/ritonavir significantly reduced viral load compared to remdesivir and control groups (*p* = 0.0117 and *p* = 0.0478). No infectious virus was detected in the lopinavir/ritonavir group, and the non-infectious SARS-CoV-2 proportion remained constant at 90%, higher than in the remdesivir and control groups (*p* = 0.0097). There was a significant difference in culture positivity among the groups (*p* = 0.0234), particularly between the lopinavir/ritonavir and remdesivir groups (*p* = 0.0267). These findings suggest that lopinavir/ritonavir reduces viral load and shortens the viral shedding duration compared to remdesivir, despite not being an effective treatment option.

## 1. Introduction

On 11 March 2020, coronavirus disease 2019 (COVID-19), which is caused by the severe acute respiratory syndrome coronavirus 2 (SARS-CoV-2), was officially categorized as a global pandemic by the World Health Organization (https://www.who.int/europe/emergencies/situations/covid-19) (accessed on 6 August 2024). This outbreak of COVID-19 led to a global health crisis, profoundly affecting medical, social, and economic frameworks [1]. In response to this unprecedented challenge, there was a significant increase in research focused on the development and deployment of antiviral drugs, such as lopinavir/ritonavir (Kaletra) and remdesivir, to alleviate the severity and duration of the infection [2,3,4,5]. The pandemic resulted in a shortage of hospital facilities, especially intensive care units, as patient numbers surged, causing healthcare systems in several countries to collapse. Non-test-based and test-based strategies were employed to determine the necessary duration of isolation and precautionary measures for individuals infected with COVID-19 [6,7].

Generally, viral shedding is more pronounced in older patients, and in those with severe symptoms, it persists in the respiratory system for up to 18 days post-symptom onset, and occasionally even months later [8]. Until May 2023, Republic of Korea recommended isolating all patients with COVID-19 for one week, regardless of their symptoms. However, the number of infectious viruses may decrease over time, meaning that long-term detection of viral RNA does not necessarily indicate long-term transmissibility [9].

Because of the rapid spread of SARS-CoV-2, there was an urgent need for effective therapeutic interventions. Although there are reports of the limited efficacy of lopinavir/ritonavir antiviral therapy in patients with COVID-19, lopinavir, as a protease inhibitor used for managing HIV infection, in combination with ritonavir, has shown promise in vitro against SARS-CoV, SARS-CoV-2, and Middle East respiratory syndrome coronavirus [2,4,10,11]. Concurrently, remdesivir, a drug originally developed for treating Ebola virus disease, has demonstrated broad-spectrum antiviral activity, including against coronaviruses [12,13]. It has been identified as a promising candidate therapeutic agent for COVID-19, owing to its ability to inhibit SARS-CoV-2 replication in vitro [14]. Despite the potential of these antiviral agents, the impact of these medications on viral shedding and transmissibility remains unclear. Notably, there is a lack of comparative studies on the duration of viral shedding in immunocompetent patients with COVID-19 receiving antiviral agents.

Our prospective observational study evaluated the efficacy of these drugs in reducing viral load and shortening the duration of viral shedding in patients with COVID-19. Specifically, we investigated the relationship between infectious viral shedding in patients treated with lopinavir/ritonavir, those treated with remdesivir, and those in the control group receiving no antiviral drugs.

## 2. Materials and Methods

### 2.1. Study Design and Patient Characteristics

This study was conducted at Chosun University Hospital between June and December 2020. Patients confirmed to be positive for SARS-CoV-2 infection either through real-time reverse transcription-polymerase chain reaction (RT-PCR) targeting at least two or more specific genes, or by demonstrating a four-fold increase in SARS-CoV-2 antibody concentrations or seroconversion, as determined by in-house indirect immunofluorescence assays [15], were enrolled in the study. Written informed consent was obtained from all the participants. All methods were approved by and performed in accordance with the relevant guidelines and regulations of the Institutional Review Board (CHOSUN 2020-04-003) of Chosun University Hospital.

The patients were divided into three groups based on the treatment received: the lopinavir/ritonavir group (*n* = 4), where patients received 400 mg lopinavir/100 mg ritonavir as a single dose on day 1 of admission to Chosun University Hospital, with treatment continued for up to 10 days; the remdesivir group (*n* = 6), where patients received a 200 mg dose of remdesivir on day 1 of admission, followed by 100 mg once daily for up to 10 days based on clinical response; and the control group (*n* = 3), where patients received no antiviral drugs. The patients’ clinical information and medical histories are presented in Table S1.

### 2.2. RNA Extraction and Real-Time Reverse Transcription-Polymerase Chain Reaction

Nasopharyngeal and oropharyngeal swabs were collected by a physician using commercial UTM kits containing 1 mL of viral transport medium (Noble Bio, Seoul, Republic of Korea) and 200 μL aliquots were used for RNA extraction. Sputum samples were self-collected by patients in collection tubes and diluted with 1 mL of phosphate-buffered saline (PBS), mixed, and centrifuged (200× *g*, 1 min). An aliquot of 200 μL of supernatant was subjected to RNA extraction. Viral RNA was extracted using a fully automated instrument (PCL, Seoul, Republic of Korea) and a Real-prep viral DNA/RNA Kit (Biosewoom, Seoul, Republic of Korea). Real-time RT-PCR was performed to target the nucleocapsid protein (*N*), envelope protein (*E*), and RNA-dependent RNA polymerase (*RdRp*) genes [16,17]. The primers (nCov-NP_572F and nCov-NP_687R) and probe (nCov-NP_661P) for the N gene were designed in-house [17]. Real-time RT-PCR targeting the *N* gene was performed using a Roche master mix (LightCycler^®^ Multiplex RNA Virus Master) in an Exicycler^TM^ 96 Real-Time Quantitative Thermal Block (Bioneer, Daejeon, Republic of Korea). The *E* and *RdRp* genes were detected using a Kogene kit (Kogene Biotech, Seoul, Republic of Korea) according to the manufacturer’s specifications and the CFX96 Touch™ Real-Time PCR Detection System (Bio-Rad, Hercules, CA, USA).

### 2.3. Cell Culture and Virus Isolation from Clinical Specimens

To isolate SARS-CoV-2 from the clinical specimens, 500 µL of sputum was mixed with 200 µL of 100× PC/SM and incubated at 4 °C for 1 h. The samples were centrifuged at 240× *g* for 20 min, and the supernatant was collected. African green monkey kidney Vero E6 cells were cultured in 24-well cell culture plates at a density of 2.5 × 10^5^ cells per well for 24 h and then inoculated with 200 µL of viral particle-containing supernatant. The plates were incubated at 37 °C, 5% CO_2_ for 3–5 days, and the infected cells were examined daily for cytopathic effects (CPE). The infected cells were then subsequently scraped from the wells, and 200 µL of the cell suspension was inoculated into a new monolayer of cultured Vero E6 cells for further incubation at 37 °C for 3–5 days (passage 1). After two passages, viral replication in the infected cells was assessed using real-time RT-PCR, and viral proliferation was confirmed by either indirect immunofluorescence assays or a real-time RT-PCR [15]. All the infection experiments were performed in a biosafety level-3 laboratory at the Health and Environment Research Institute of Gwangju City.

### 2.4. Whole-Genome Sequencing of SARS-CoV-2 Isolates

Whole-genome sequences of the SARS-CoV-2 isolates were obtained using an Illumina MiSeq next-generation sequencer with 150PE (San Diego, CA, USA) and assembled using Bowtie v.1.1.2 and samtools-mpileup (samtools v.1.9) software [16]. The complete genome sequences of the SARS-CoV-2 isolates were registered with the Global Initiative for Sharing All Influenza Data (GISAID).

### 2.5. Phylogenetic Analysis

Phylogenetic trees were constructed based on complete genome sequences of SARS-CoV-2 isolates generated in this study and from reference sequences retrieved from GISAID. ClustalX (version 2.0; http://www.clustal.org/) (accessed on 6 August 2024) and Tree Explorer (DNASTAR) were used to construct the phylogenetic trees. To increase the reliability of the tree, a bootstrap analysis was conducted with 1000 replicates.

### 2.6. Indirect Immunofluorescence Assay

For indirect immunofluorescence assays (IFA), each patient’s serum was diluted using a two-fold serial dilution from 1:16 and then reacted with the SARS-CoV-2 antigen slide in a moist chamber for 30 min at 37 °C, as described previously [15]. To prepare a SARS-CoV-2 antigen slide, Vero E6 cells infected with the SARS-CoV-2 virus for 3 days were cultured on Teflon-coated multiwell slides overnight at 37 °C and 5% CO_2_ and were fixed with 80% acetone the next day. After washing, the slides were incubated with secondary antibodies (1:400 dilution; fluorescein isothiocyanate-conjugated anti-human IgM and IgG; MP Biomedicals, Irvine, CA, USA). The slides were examined under a fluorescence microscope (Olympus IX73, Olympus, Tokyo, Japan, magnification: 400×) after dispensing the mounting solution (Vector Laboratories, Burlingame, CA, USA). IgG and IgM antibody titers ≥ 1:32 were selected as the positive cutoff values based on the results from 15 health check-up participants.

### 2.7. Statistical Analysis

Statistical analyses were performed using GraphPad prism, version 8.0 (Boston, MA, USA) and IBM SPSS Statistics for Windows, version 26.0. (IBM Corp., Armonk, NY, USA). The Kruskal–Wallis test was used to assess differences between the three groups (lopinavir/ritonavir, remdesivir, and no antiviral treatment) for both the upper and lower respiratory tract data. A post hoc analysis was performed using Tukey’s multiple comparisons test to identify statistically significant differences between each pair of groups on each day. The Mann–Whitney U test was applied to compare Ct values and viral loads between culture-positive and culture-negative samples. The significance of culture results was evaluated using the Kruskal–Wallis test. Additionally, a Kaplan–Meier survival analysis was conducted to compare the effectiveness of lopinavir/ritonavir or remdesivir with no antiviral treatment, as well as survival up to 20 days. A *p*-value < 0.05 was considered to be statistically significant for all tests.

## 3. Results

Patients positive for SARS-CoV-2 infection were divided into three groups: those treated with lopinavir/ritonavir (*n* = 4), those treated with remdesivir (*n* = 6), and a control group receiving no antiviral drugs (*n* = 3). Three patients, identified through contact tracing, exhibited upper respiratory tract infection symptoms without pneumonia and were isolated without antiviral treatment. Four patients in the lopinavir/ritonavir group received the antiviral treatment for up to 10 days, whereas six patients in the remdesivir group received antiviral treatment based on the clinical response.

Antiviral activity, viral infectivity, and clearance among patients were compared across the three groups. Real-time RT-PCR was performed on samples from patients to determine the inhibition rate of SARS-CoV-2 replication by lopinavir/ritonavir and remdesivir. Infectious viral shedding during hospitalization was confirmed using Vero E6 cell cultures (Table S2).

The presence or absence of infectious SARS-CoV-2 during hospitalization and the cycle threshold (Ct) values of real-time RT-PCR from all serial respiratory samples (nasopharyngeal and oropharyngeal swabs and sputum samples) were compared (Figure S1). SARS-CoV-2 was cultivated from 50 of the 110 cultured samples (45.5%) obtained from patients with COVID-19. Infectious viruses were detected only in samples with Ct values ≤ 35, with a median Ct value of 19.38 for the *RdRp* gene-targeted real-time RT-PCR (Figure S1A). The mean viral load in culture-positive samples was significantly higher than in culture-negative samples. SARS-CoV-2 was isolated from 14 of the 35 cultured samples (40.0%) obtained from patients administered with remdesivir; infectious viruses were detected only in samples with Ct values ≤ 29 (Figure S1B), with a median Ct value of 15.57. In patients with severe COVID-19 who were treated with lopinavir/ritonavir, no infectious SARS-CoV-2 was detected after administration (Figure S1C). Samples from patients with mild symptoms who did not receive antiviral agents were also tested using the *RdRp* gene-targeted real-time RT-PCR and cultured to detect SARS-CoV-2 infection. The virus was isolated from 31 of the 43 cultured samples and continuously cultivated for up to eight days after symptom onset (eight days post-hospitalization) (Figure S1D).

**Figure 1:**
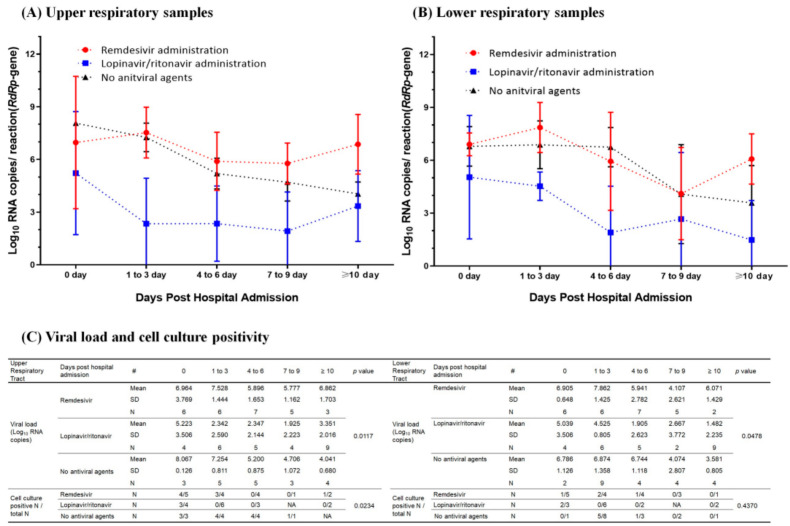
SARS-CoV-2 viral loads determined using *RdRp* gene-targeted real-time reverse transcription-polymerase chain reaction in upper respiratory (nasopharyngeal swab) (**A**) and lower respiratory (sputum) (**B**) tract samples. Viral load and cell culture positivity showing infectious viral shedding in upper and lower respiratory tract samples (**C**) were obtained from patients treated with remdesivir, lopinavir/ritonavir, or no antiviral drugs. N, number of samples; NA, not assayed.

Throughout the hospitalization period, the lopinavir/ritonavir group exhibited a consistently lower viral load in both the upper and lower respiratory tract samples than compared to the remdesivir group and the control group. The remdesivir group exhibited a higher or comparable mean viral load than compared to the control group (Figure 1A,B). All nasopharyngeal swab samples from patients not receiving antiviral agents were cultured for up to 8 days during hospitalization. Three of four nasopharyngeal swab samples were culture-positive for patients administered lopinavir/ritonavir on the day of hospitalization (day 0). However, all subsequent cultures from the lopinavir/ritonavir-treated group tested negative. Conversely, in patients administered remdesivir, four out of five nasopharyngeal swab samples showed positive culture results on day 0 of hospitalization. During days 1–3 of hospitalization, three out of four samples were culture-positive, with one remaining culture-positive even on day 12. Significant differences in viral loads were observed among the three groups in both upper (nasopharyngeal swab samples) and lower (sputum) respiratory tract samples (*p* = 0.0117 and *p* = 0.0478, respectively), and in culture positivity among nasopharyngeal swab samples (*p* = 0.0234), specifically between the lopinavir/ritonavir and remdesivir groups (*p* = 0.0267) (Figure 1C).

**Figure 2:**
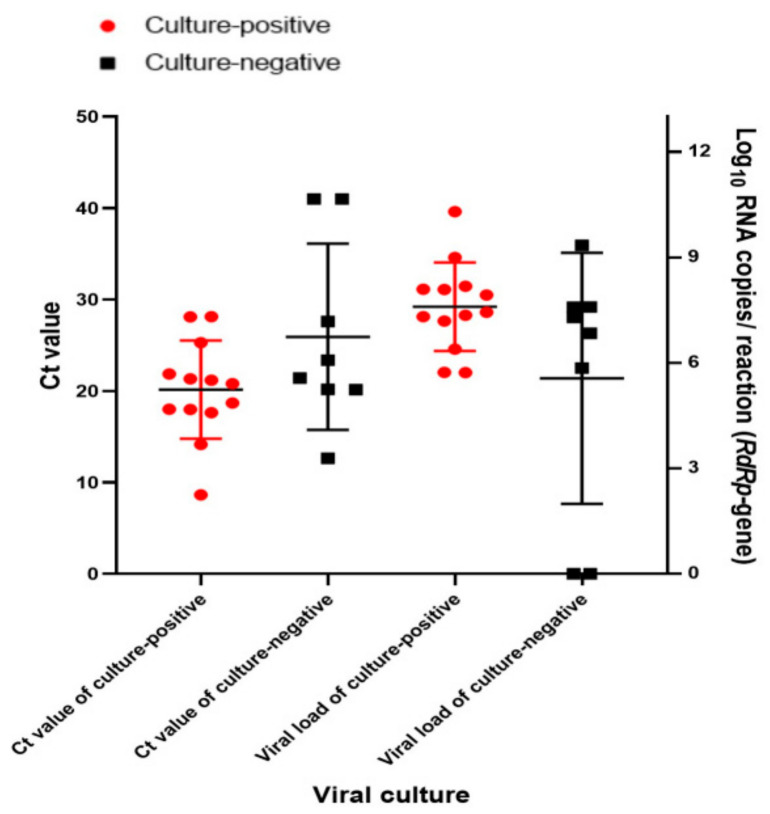
Log_10_ RNA copies/reaction (*RdRp*-gene) and Ct value of culture-positive (red circles) and culture-negative (black squares) samples, including nasopharyngeal swab samples and sputum samples, from patients with COVID-19 on hospitalization day (day 0).

We compared infectious viral shedding, as determined by cell culture, and the Ct values of *RdRp* gene-targeted real-time RT-PCR of the upper and lower respiratory tract samples on the day of hospitalization (day 0). The positivity rate for viral cultures on day 0 was 61.9% (13 of 21 cases). The median Ct value and the mean viral load of culture-positive samples on day 0 were 20.08 and 7.59 × Log_10_ RNA copies/reaction, respectively. The mean viral copy number of culture-positive samples on day 0 (3.93 × 10^7^ RNA copies) was higher than that of culture-negative samples (3.66 × 10^5^ RNA copies) (Figure 2).

Additionally, the proportion of non-infectious SARS CoV-2 remained constant at 90% for the lopinavir/ritonavir-treated group, higher than that of the remdesivir-treated and non-treated groups (*p* = 0.0097). According to the Kaplan–Meier curve, the remdesivir-treated group exhibited a positive culture 24 h after drug administration. In contrast, the lopinavir/ritonavir-treated group showed no positive culture 24 h after drug administration (Figure S2).

NGS sequencing was conducted on viral RNA extracted from cell culture supernatants of seven nasopharyngeal samples. Phylogenetic analysis was performed on seven SARS-CoV-2 whole genome sequences from this study and several reference sequences obtained from the GISAID database. Among the analyzed SARS-CoV-2 genome sequences, four SARS-CoV-2 isolates were classified into the PANGO lineage B and GISAID clade V, whereas three isolates were identified as belonging to PANGO lineage B.1.497 and GISAID clade GH (Figure S3).

## 4. Discussion

The inaccuracies of PCR tests in managing COVID-19 underscore the delicate balance between public health and resource allocation, emphasizing the critical need for accurate diagnostics to devise effective strategies. False-negative PCR results can lead to insufficient isolation of infectious individuals, potentially causing wider virus spread [18]. Conversely, false-positive results may unnecessarily alarm healthcare workers and lead to the wasteful isolation and testing of contacts who are not at risk [19]. In our study, viral cultures and Ct values were used to further investigate the presence of infectious SARS-CoV-2, providing additional insights into viral load and shedding patterns. Viral cultures confirmed infectious viral shedding during hospitalization, with infectious viruses detected only in samples with Ct values ≤ 35 and a median Ct value of 19.38 for the *RdRp* gene-targeted real-time RT-PCR. This correlation between Ct values and viral infectivity helps explain the occurrence of false-negatives and -positives. Samples with high Ct values (indicating low viral loads) may still test positive with PCR but not be infectious, while those with low Ct values are more likely to be infectious.

A Chinese in Taiwan study involving 2500 close contacts of 100 patients with COVID-19 revealed that all 22 secondary infections occurred within six days of symptom onset in the initial case, with none of the 850 contacts exposed afterwards becoming infected [20]. In contrast, a Chinese in Mainland study reported that COVID-19 transmission could start as early as 2.3 days before symptom onset, with peak transmissibility 0.7 days before symptom onset and a gradual decline thereafter [21]. According to the CDC, it is rare to isolate infectious viruses from immunocompetent patients 7–10 days after symptom onset, thereby recommending that precautions and isolation be discontinued after 10 days [22]. Nonetheless, replication-competent viruses have been detected in respiratory or fecal samples from patients with COVID-19 who are severely ill, critically ill, or immunocompromised more than 10 days post-symptom onset [23,24]. During the pandemic in Republic of Korea, all patients with COVID-19, whether symptomatic or asymptomatic, were isolated in negative pressure rooms or community treatment centers [9,25]. Test-based strategies initially required discontinuing isolation only after two negative real-time PCR tests conducted at least 24 h apart. Nevertheless, the prolonged detection of viral RNA has not been linked to prolonged transmissibility [9].

Our study contributes to understanding the effects of lopinavir/ritonavir and remdesivir on SARS-CoV-2 viral shedding in patients with COVID-19. The patients that were confirmed positive for SARS-CoV-2 through RT-PCR or seroconversion were divided into three groups: those treated with lopinavir/ritonavir, those with remdesivir, and a control group without antiviral drugs. We observed that in patients with mild symptoms who were not treated with either antiviral, the virus could be detected for up to 10 days after symptom onset or hospital admission. Interestingly, no infectious viruses were detected in specimens from patients administered with lopinavir/ritonavir, whereas a few infectious viruses were detected in specimens from patients administered with remdesivir. Our investigation reveals notable differences in viral load and clearance among these groups, underscoring the potential efficacy of antiviral regimens in managing COVID-19. Furthermore, this study demonstrates a lower viral load and faster viral clearance in the lopinavir/ritonavir group compared to those receiving remdesivir or no treatment. These results are significant, as no previous studies have compared lopinavir/ritonavir and remdesivir treatments from the perspective of virus culture.

The antiviral effect of lopinavir/ritonavir against the SARS-CoV-2 virus has been reported in several studies. Lopinavir/ritonavir has a significant inhibitory effect on SARS-CoV-2, indicating its potential as a therapeutic agent against the virus [26]. However, a previous open-label randomized trial indicated that a 10-day regimen of lopinavir/ritonavir did not markedly influence 28-day mortality rates or the need for mechanical ventilation in hospitalized patients with COVID-19 [11]. In addition, despite reports of high plasma lopinavir concentrations in patients with COVID-19, the concentration of unbound drugs in the lungs is insufficient to inhibit SARS-CoV-2 replication [27,28]. Moreover, some studies have reported a poor response to lopinavir/ritonavir antiviral therapy in patients with COVID-19, indicating its limited therapeutic efficacy [29]. Therefore, lopinavir/ritonavir administration may not be appropriate for therapeutic purposes. In contrast, our study demonstrates that a significant inhibitory effect of lopinavir/ritonavir, even after a single dose, is more effective than remdesivir in reducing viral load and shortening the viral shedding period in patients with COVID-19.

The evolution of SARS-CoV-2 and the emergence of new variants underscore the need for ongoing evaluation of antiviral agents. The specimens obtained in the present study primarily consisted of variants from early in the pandemic in the Republic of Korea, such as PANGO lineage B and PANGO lineage B.1.497. By the end of 2020, the original L strain of the virus had undergone multiple mutations, leading to the emergence of the S, V, and G strains. Since the discovery of the alpha variant (B.1.1.7) in the United Kingdom in September 2020, several variants have emerged, including beta (B.1.351), gamma (P.1), and delta (B.1.617.2) variants, and, in late November 2021, the original omicron variant (B.1.1.529), followed by several subvariants (https://www.verywellhealth.com/covid-variants-timeline-6741198) (accessed on 6 August 2024).

It is important to note that our study’s results should be interpreted with caution. The lack of previous comparative studies between lopinavir/ritonavir and remdesivir highlights the novelty of our study, but also suggests the need for further studies to validate our findings. Moreover, considering the evolving nature of the COVID-19 pandemic and the emergence of new variants, the effectiveness of these antiviral agents might vary and should be continuously evaluated. Limitations of this study include its small sample size from a single institution. Among patients treated with lopinavir/ritonavir and remdesivir, only ten underwent culture testing, and drug plasma concentrations were not monitored. Patients treated with lopinavir/ritonavir showed significantly fewer viral RNA copies and were more culture-negative than those treated with remdesivir. A report on the adverse effects associated with remdesivir, hydroxychloroquine, and lopinavir/ritonavir in patients with COVID-19 indicates that lopinavir/ritonavir may increase the risk of gastrointestinal adverse events, including diarrhea, nausea, and vomiting, compared to standard care or placebo [30]. These findings highlight the importance of considering potential side effects when evaluating the efficacy of antiviral medications.

Considering the potential influence of age and severity on the effectiveness of each treatment, additional studies are necessary to examine their impact on infectious viral shedding. Further research is also required to understand the effects and mechanisms of action of lopinavir/ritonavir on COVID-19 transmissibility across different variants. It is crucial to investigate appropriate dosages for the short-term administration of lopinavir/ritonavir and remdesivir to avoid inducing viral resistance and to assess potential side effects. Moreover, exploring combination therapies involving antivirals and immune-modulating compounds could provide insights into more effective treatment strategies for COVID-19. For example, the combination of remdesivir with immune-modulating agents such as p38 inhibitors, baricitinib, and host-directed drugs such as FIASMAs or itraconazole has shown promise [31,32,33]. These findings suggest that such combinatory treatments could enhance antiviral efficacy by targeting multiple pathways involved in viral replication and immune response [34]. Moreover, it is important to note that remdesivir is less stable in the blood of patients; however, remdesivir metabolite GS-441524 is more stable and thus may be a better option for the treatment of viral infections [35]. Notably, GS-441524 showed a similar synergy when administered with the antidepressant fluoxetine, highlighting its potential clinical benefit over remdesivir [36]. Therefore, our study offers valuable clinical research data, highlighting the importance of further investigations into combination therapies and alternative antiviral agents such as GS-441524 to optimize COVID-19 treatment.

In conclusion, we show that patients with COVID-19 and who were administered lopinavir/ritonavir may show lower infectious viral shedding than those administered with remdesivir or no antiviral drugs.

## Data Availability

The datasets generated during the current study are available in the data repository of the journal.

## Supplementary Materials

The following supporting information can be downloaded from: https://www.mdpi.com/article/10.3390/microorganisms12081696/s1. Figure S1. Detection of SARS-CoV-2 RNA viral load via *RdRp* gene-targeted real-time reverse transcription-polymerase chain reaction and identification of infectious virus particles in Vero E6 cell cultures from clinical specimens. (A) All serial respiratory samples (nasopharyngeal and oropharyngeal swabs and sputum samples) obtained from patients diagnosed with COVID-19, (B) serial respiratory samples obtained from patients treated with remdesivir during their hospital stay, (C) serial respiratory samples obtained from patients treated with lopinavir/ritonavir during their hospital stay, and (D) serial respiratory samples obtained from patients not treated with any antiviral agents during their hospital stay. Figure S2. Kaplan–Meier curves showing the proportion of non-infectious SARS-CoV-2 in clinical samples from the lopinavir/ritonavir group, the remdesivir group, and the group not treated with antiviral agents. Figure S3. Phylogenetic tree constructed from SARS-CoV-2 complete genome sequences of seven SARS-CoV-2-positive culture supernatants from this study, as well as several reference sequences from the GISAID database. The tree is drawn to scale, with branch lengths representing the number of substitutions per site. To assess the confidence of the tree, 1000 bootstrap replicates were performed. The GISAID accession numbers for the seven SARS-CoV-2 isolates are provided. PANGO lineage and GISAID clades are indicated on the right. Table S1. Clinical information of patients with COVID-19 who were not treated with any antiviral drug and those who were treated with remdesivir or lopinavir/ritonavir as an antiviral drug. Table S2. Real-time PCR and viral culture results of patients with COVID-19 who were not treated with any antiviral drug and those who were treated with remdesivir or lopinavir/ritonavir as an antiviral drug.

## References

[1] Kneip T., Borsch-Supan A., Andersen-Ranberg K. (2022). Social, health and economic impact of the COVID-19 pandemic from a European perspective. Eur. J. Ageing.

[2] Baldelli S., Corbellino M., Clementi E., Cattaneo D., Gervasoni C. (2020). Lopinavir/ritonavir in COVID-19 patients: Maybe yes, but at what dose?. J. Antimicrob. Chemother..

[3] Hassaniazad M., Bazram A., Hassanipour S., Fathalipour M. (2020). Evaluation of the efficacy and safety of favipiravir and interferon compared to lopinavir/ritonavir and interferon in moderately ill patients with COVID-19: A structured summary of a study protocol for a randomized controlled trial. Trials.

[4] Chen C., Fang J., Chen S., Rajaofera M.J.N., Li X., Wang B., Xia Q. (2023). The efficacy and safety of remdesivir alone and in combination with other drugs for the treatment of COVID-19: A systematic review and meta-analysis. BMC Infect. Dis..

[5] Tasavon Gholamhoseini M., Yazdi-Feyzabadi V., Goudarzi R., Mehrolhassani M.H. (2021). Safety and Efficacy of Remdesivir for the Treatment of COVID-19: A Systematic Review and Meta-Analysis. J. Pharm. Pharm. Sci..

[6] World Health Organization (2020). Criteria for Releasing COVID-19 Patients from Isolation.

[7] Centers for Disease Control and Prevention (2021). Ending Isolation and Precautions for People with COVID-19: Interim Guidance.

[8] Fontana L.M., Villamagna A.H., Sikka M.K., McGregor J.C. (2021). Understanding viral shedding of severe acute respiratory coronavirus virus 2 (SARS-CoV-2): Review of current literature. Infect. Control Hosp. Epidemiol..

[9] Wolfel R., Corman V.M., Guggemos W., Seilmaier M., Zange S., Muller M.A., Niemeyer D., Jones T.C., Vollmar P., Rothe C. (2020). Virological assessment of hospitalized patients with COVID-2019. Nature.

[10] Cvetkovic R.S., Goa K.L. (2003). Lopinavir/ritonavir: A review of its use in the management of HIV infection. Drugs.

[11] Group R.C. (2020). Lopinavir-ritonavir in patients admitted to hospital with COVID-19 (RECOVERY): A randomised, controlled, open-label, platform trial. Lancet.

[12] Eastman R.T., Roth J.S., Brimacombe K.R., Simeonov A., Shen M., Patnaik S., Hall M.D. (2020). Remdesivir: A Review of Its Discovery and Development Leading to Emergency Use Authorization for Treatment of COVID-19. ACS Cent. Sci..

[13] Yan D., Ra O.H., Yan B. (2021). The nucleoside antiviral prodrug remdesivir in treating COVID-19 and beyond with interspecies significance. Anim. Dis..

[14] Beigel J.H., Tomashek K.M., Dodd L.E., Mehta A.K., Zingman B.S., Kalil A.C., Hohmann E., Chu H.Y., Luetkemeyer A., Kline S. (2020). Remdesivir for the Treatment of Covid-19-Final Report. N. Engl. J. Med..

[15] Lawrence Panchali M.J., Kim C.M., Seo J.W., Kim D.Y., Yun N.R., Kim D.M. (2023). SARS-CoV-2 RNAemia and Disease Severity in COVID-19 Patients. Viruses.

[16] Chatterjee S., Kim C.M., Lee Y.M., Seo J.W., Kim D.Y., Yun N.R., Kim D.M. (2022). Whole-genome analysis and mutation pattern of SARS-CoV-2 during first and second wave outbreak in Gwangju, Republic of Korea. Sci. Rep..

[17] Lawrence Panchali M.J., Oh H.J., Lee Y.M., Kim C.M., Tariq M., Seo J.W., Kim D.Y., Yun N.R., Kim D.M. (2022). Accuracy of Real-Time Polymerase Chain Reaction in COVID-19 Patients. Microbiol. Spectr..

[18] Woloshin S., Patel N., Kesselheim A.S. (2020). False Negative Tests for SARS-CoV-2 Infection—Challenges and Implications. N. Engl. J. Med..

[19] Jia X., Xiao L., Liu Y. (2021). False negative RT-PCR and false positive antibody tests-Concern and solutions in the diagnosis of COVID-19. J. Infect..

[20] Cheng H.Y., Jian S.W., Liu D.P., Ng T.C., Huang W.T., Lin H.H., The Taiwan COVID-19 Outbreak Investigation Team (2020). Contact Tracing Assessment of COVID-19 Transmission Dynamics in Taiwan and Risk at Different Exposure Periods Before and After Symptom Onset. JAMA Intern. Med..

[21] He X., Lau E.H.Y., Wu P., Deng X., Wang J., Hao X., Lau Y.C., Wong J.Y., Guan Y., Tan X. (2020). Temporal dynamics in viral shedding and transmissibility of COVID-19. Nat. Med..

[22] Jernigan D.B. (2020). Update: Public Health Response to the Coronavirus Disease 2019 Outbreak—United States, February 24, 2020.

[23] Choi B., Choudhary M.C., Regan J., Sparks J.A., Padera R.F., Qiu X., Solomon I.H., Kuo H.H., Boucau J., Bowman K. (2020). Persistence and Evolution of SARS-CoV-2 in an Immunocompromised Host. N. Engl. J. Med..

[24] Avanzato V.A., Matson M.J., Seifert S.N., Pryce R., Williamson B.N., Anzick S.L., Barbian K., Judson S.D., Fischer E.R., Martens C. (2020). Case Study: Prolonged Infectious SARS-CoV-2 Shedding from an Asymptomatic Immunocompromised Individual with Cancer. Cell.

[25] Kang E., Lee S.Y., Jung H., Kim M.S., Cho B., Kim Y.S. (2020). Operating Protocols of a Community Treatment Center for Isolation of Patients with Coronavirus Disease, South Korea. Emerg. Infect. Dis..

[26] Kang C.K., Seong M.W., Choi S.J., Kim T.S., Choe P.G., Song S.H., Kim N.J., Park W.B., Oh M.D. (2020). In vitro activity of lopinavir/ritonavir and hydroxychloroquine against severe acute respiratory syndrome coronavirus 2 at concentrations achievable by usual doses. Korean J. Intern. Med..

[27] Gregoire M., Le Turnier P., Gaborit B.J., Veyrac G., Lecomte R., Boutoille D., Canet E., Imbert B.M., Bellouard R., Raffi F. (2020). Lopinavir pharmacokinetics in COVID-19 patients. J. Antimicrob. Chemother..

[28] Marzolini C., Stader F., Stoeckle M., Franzeck F., Egli A., Bassetti S., Hollinger A., Osthoff M., Weisser M., Gebhard C.E. (2020). Effect of Systemic Inflammatory Response to SARS-CoV-2 on Lopinavir and Hydroxychloroquine Plasma Concentrations. Antimicrob. Agents Chemother..

[29] Osborne V., Davies M., Lane S., Evans A., Denyer J., Dhanda S., Roy D., Shakir S. (2020). Lopinavir-Ritonavir in the Treatment of COVID-19: A Dynamic Systematic Benefit-Risk Assessment. Drug Saf..

[30] Izcovich A., Siemieniuk R.A., Bartoszko J.J., Ge L., Zeraatkar D., Kum E., Qasim A., Khamis A.M., Rochwerg B., Agoritsas T. (2022). Adverse effects of remdesivir, hydroxychloroquine and lopinavir/ritonavir when used for COVID-19: Systematic review and meta-analysis of randomised trials. BMJ Open.

[31] Faist A., Schloer S., Mecate-Zambrano A., Janowski J., Schreiber A., Boergeling Y., Conrad B.C.G., Kumar S., Toebben L., Schughart K. (2023). Inhibition of p38 signaling curtails the SARS-CoV-2 induced inflammatory response but retains the IFN-dependent antiviral defense of the lung epithelial barrier. Antivir. Res..

[32] Kalil A.C., Patterson T.F., Mehta A.K., Tomashek K.M., Wolfe C.R., Ghazaryan V., Marconi V.C., Ruiz-Palacios G.M., Hsieh L., Kline S. (2021). Baricitinib plus Remdesivir for Hospitalized Adults with Covid-19. N. Engl. J. Med..

[33] Schloer S., Brunotte L., Mecate-Zambrano A., Zheng S., Tang J., Ludwig S., Rescher U. (2021). Drug synergy of combinatory treatment with remdesivir and the repurposed drugs fluoxetine and itraconazole effectively impairs SARS-CoV-2 infection in vitro. Br. J. Pharmacol..

[34] Kojima Y., Nakakubo S., Kamada K., Yamashita Y., Takei N., Nakamura J., Matsumoto M., Horii H., Sato K., Shima H. (2022). Combination therapy with remdesivir and immunomodulators improves respiratory status in COVID-19: A retrospective study. J. Med. Virol..

[35] Schooley R.T., Carlin A.F., Beadle J.R., Valiaeva N., Zhang X.Q., Clark A.E., McMillan R.E., Leibel S.L., McVicar R.N., Xie J. (2021). Rethinking Remdesivir: Synthesis, Antiviral Activity, and Pharmacokinetics of Oral Lipid Prodrugs. Antimicrob. Agents Chemother..

[36] Brunotte L., Zheng S., Mecate-Zambrano A., Tang J., Ludwig S., Rescher U., Schloer S. (2021). Combination Therapy with Fluoxetine and the Nucleoside Analog GS-441524 Exerts Synergistic Antiviral Effects against Different SARS-CoV-2 Variants In Vitro. Pharmaceutics.

