# Supplementary material for: Comparable Efficacy of Lopinavir/Ritonavir and Remdesivir in Reducing Viral Load and Shedding Duration in Patients with COVID-19
Source: Microorganisms. 2024 Aug 16;12(8):1696. doi: 10.3390/microorganisms12081696 (PMC11357406; doi:10.3390/microorganisms12081696)
Supplement: Supplementary file 1 [file microorganisms-12-01696-s001.zip › Supplementary Tables S1 and S2-MDPI.pdf]

Table S1. Clinical information of patients with COVID-19 who were not administrated any antiviral drug and patients with COVID-19 who were administrated Remdesivir, Lopinavir/ritonavir as an antiviral drug*.

Antiviral agents	Patients no.	Day 1	Day 2	Age (Years)	Gender	Symptoms	O_2 inhalation	Mechanical ventilation	Pneumonia	Steroids	Medication date	Blood collection date	Underlying other diseases
No antiviral drug	#1	20-Feb	0	30	Male	coughing, sore throat chillness	N	N	N	N	-	-	HTN
No antiviral drug	#2	21-Feb	0	30	Male	febrile sense	N	N	N	N	-	-	-
No antiviral drug	#3	23-Feb	1	29	Female	sore throat, myalgia chillness	N	N	N	N	-	-	-
Remdesivir	#1	2-Jan	9	83	Male	blood Pressure decrease, O2 rate decrease	Y	N	Y	Y	2-Jan 13:00	2-Jan<br>3-Jan	HTN, CVA, dementia
Remdesivir	#2	1-Jul	3	56	Male	febrile sense, diarrhea	Y	N	Y	N	6-Jul 20:30	6-Jul 05:05<br>8-Jul 05:16	HTN, colon cancer
Remdesivir	#3	3-Jul	0	93	Female	myalgia	Y	N	Y	N	6-Jul 20:30	6-Jul 05:16<br>9-Jul 05:12	HTN, MDD
Remdesivir	#4	18-Jul	12	69	Male	cough, myalgia	Y	Y	Y	Y	19-Jul 11:00	19-Jul 05:06<br>20-Jul 05:36	DM, BPH
Remdesivir	#5	28-Aug	1	89	Female	fever	Y	N	Y	N	28-Aug 17:10	28-Aug 05:28<br>31-Aug 05:40	Dementia
Remdesivir	#6	27-Dec	0	87	Female	blood Pressure decrease, O2 rate decrease	Y	N	Y	Y	27-Dec 17:00	27-Dec<br>28-Dec	HTN, DM, Dementia
Lopinavir/ritonavir	#1	6-Feb	-2	46	Male	coughing, chillness	N	N	N	N	6-Feb 21:19	6-Feb 11:50<br>10-Feb 07:49	HTN, dyslipidemia
Lopinavir/ritonavir	#2	2-Mar	7	74	Male	sore throat, rhinorrhea	N	N	N	N	2-Mar 20:48	2-Mar 16:25<br>5-Mar 08:26	HTN, DM, BPH
Lopinavir/ritonavir	#3	28-Feb	1	79	Female	dyspnea	Y	Y	N	Y	28-Feb 01:40	28-Feb 02:54<br>29-Feb 10:02	HTN, DM, Guillain-Barre syndrome
Lopinavir/ritonavir	#4	10-Mar	9	79	Male	fever, Headache	Y	Y	N	Y	11-Mar 08:00	10-Mar 19:03<br>11-Mar 08:20	HTN, DM

* Day 1, Hospitalization day; Day 2, days after symptom onset; N, No; Y, Yes; HTN, Hypertension; CVA, cerebrovascular accident; MDD, major depressive disorder; DM, diabetes mellitus, BPH, benign prostatic hypertrophy

Table S2. The Real-time PCR and viral culture results of patients with COVID-19 who were not administrated any antiviral drug and patients with COVID-19 who were administrated Remdesivir or Lopinavir/ritonavir as an antiviral drug*.

Antiviral agents	Pat. no.	Date	Day 1	Day 2	**Upper Airway (**Nasopharyngeal swab samples)							**Lower Airway** (Sputum samples)						
					**Ct value (N-gene)**	**Viral RNA Copies**	**Ct value (E-gene)**	**Viral RNA Copies**	**Ct value (RdRP-gene)**	**Viral RNA Copies**	**Viral Culture**	**Ct value (N-gene)**	**Viral RNA Copies**	**Ct value (E-gene)**	**Viral RNA Copies**	**Ct value (RdRP-gene)**	**Viral RNA Copies**	**Viral Culture**
No antiviral drug	#1	20-Feb	#0	0	18.84	4.53E+08	17.96	8.01E+07	18.02	1.23E+08	P	20.79	1.34E+08	20.20	1.70E+07	20.19	3.83E+07	N
		21-Feb	#1	1	17.85	8.42E+08	17.74	9.32E+07	17.88	1.33E+08	P	25.83	5.70E+06	25.82	3.48E+05	26.25	1.47E+06	P
		22-Feb	#2	2	NA		NA		NA			18.64	5.13E+08	18.53	5.40E+07	18.7	8.55E+07	N
		23-Feb	#3	3	NA		NA		NA			32.14	1.10E+05	33.70	1.49E+03	32.06	6.46E+04	N
		24-Feb	#4	4	27.47	2.04E+06	27.54	1.06E+05	27.69	6.78E+05		30.5	3.06E+05	30.64	1.24E+04	30.53	1.47E+05	N
		25-Feb	#5	5	31.44	1.70E+05	31.83	5.43E+03	31.91	7.00E+04	P	NA		NA		NA		
		26-Feb	#6	6	26.48	3.79E+06	25.40	4.65E+05	25.55	2.14E+06	P	NA		NA		NA		
		28-Feb	#8	8	29.3	6.49E+05	28.40	5.83E+04	28.76	3.81E+05	P	NA		NA		NA		
		29-Feb	#9	9	NA		NA		NA			32.76	7.44E+04	32.55	3.30E+03	32.61	4.80E+04	N
		02-Mar	#11	11	31.83	1.33E+05	31.55	6.60E+03	32.04	6.53E+04		36.16	8.86E+03	UD	0	UD	0	N
	#2	21-Feb	#0	0	20.70	1.41E+08	16.19	2.73E+08	17.63	1.52E+08	P	17.24	1.23E+09	13.76	1.46E+09	NA		
		22-Feb	#1	1	NA		NA		NA			17.31	1.18E+09	17.83	8.76E+07	14.17	9.78E+08	P
		23-Feb	#2	2	NA		NA		NA			27.41	2.12E+06	29.61	2.53E+04	18.01	1.24E+08	P
		24-Feb	#3	3	25.05	9.29E+06	25.26	5.12E+05	26.04	1.65E+06		NA		NA		28.92	3.50E+05	P
		25-Feb	#4	4	23.92	1.88E+07	NA		NA			NA		NA		NA		
		26-Feb	#5	5	23.28	2.81E+07	32.31	3.90E+03	31.8	7.43E+04	P	20.26	1.86E+08	23.88	1.33E+06	NA		
		27-Feb	#6	6	NA		NA		NA			23.69	2.18E+07	24.76	7.24E+05	21.87	1.55E+07	N
		28-Feb	#7	7	23.65	2.23E+07	30.35	1.51E+04	31.12	1.07E+05		28.76	9.10E+05	30.07	1.84E+04	25.31	2.44E+06	N
		29-Feb	#8	8	NA		NA		NA			NA		NA		30.27	1.69E+05	
		02-Mar	#10	10	NA		33.28	1.99E+03	33.87	2.44E+04		NA		NA		NA		
		04-Mar	#12	12	31.68	1.46E+05	35.03	5.93E+02	37.1	4.29E+03		27.39	2.15E+06	29.06	3.69E+04	29.67	2.34E+05	
		05-Mar	#13	13	NA		NA		NA			34.75	2.14E+04	36.38	2.33E+02	35.4	1.07E+04	
		06-Mar	#14	14	UD	0	UD	0	38.39	2.14E+03		33.64	4.29E+04	36.62	1.97E+02	37.23	4.00E+03	
	#3	23-Feb	#0	1	16.56	1.89E+09	18.35	6.11E+07	18.70	8.55E+07	P	NA		27.27	1.28E+05	27.01	9.78E+05	
		24-Feb	#1	2	17.31	1.18E+09	19.08	3.69E+07	19.27	6.29E+07	P	23.74	2.11E+07	27.31	1.24E+05	26.98	9.93E+05	P
		25-Feb	#2	3	19.10	3.85E+08	20.30	1.59E+07	20.40	3.42E+07	P	NA		NA		20.60	3.07E+07	N
		26-Feb	#3	4	22.27	5.29E+07	24.21	1.06E+06	24.40	3.98E+06	P	21.14	1.07E+08	22.83	2.75E+06	23.34	7.04E+06	
		28-Feb	#5	6	32.31	9.86E+04	34.33	9.63E+02	35.00	1.33E+04	P	NA		NA		19.38	5.93E+07	P
		2-Mar	#8	9	34.26	2.91E+04	37.09	1.43E+02	37.62	3.24E+03		NA		NA		NA		
		3-Mar	#9	10	NA		NA		NA			UD	0	UD	0	UD	0	
		4-Mar	#10	11	UD	0	NA		NA			30.08	3.98E+05	31.14	8.76E+03	31.65	8.05E+04	
		6-Mar	#12	13	UD	0	NA		NA			NA		NA		NA		
Remdesivir	#1	2-Jan	#0	9			8.49	5.62E+10	8.65	1.91E+10	P			27.83	8.65E+04	27.6	7.12E+05	N
		4-Jan	#2	11			12.53	3.43E+09	11.35	4.46E+09	P			13.9	1.33E+09	12.96	1.87E+09	N
		7-Jan	#5	14			16.65	1.98E+08	16.34	3.04E+08	N			15.93	3.26E+08	15.09	5.96E+08	P
		11-Jan	#9	18			21.06	9.37E+06	20.93	2.57E+07				23.83	1.38E+06	23.32	7.12E+06	
		14-Jan	#12	21			15.58	4.16E+08	15.79	4.09E+08	P							
	#2	1-Jul	#0	3	UD	0	36.02	2.99E+02	UD	0				24.36	9.55E+05	24.84	3.14E+06	
		3-Jul	#2	5			25.58	4.11E+05	27.26	8.55E+05				28.13	7.03E+04	28.39	4.65E+05	
		6-Jul	#5	8			23.31	1.98E+06	24.11	4.65E+06	N			25.69	3.81E+05	26.16	1.54E+06	N

		8-Jul	#7	10			25.02	6.05E+05	26.1	1.59E+06				31.4	7.32E+03	34.92	1.39E+04	N
		10-Jul	#9	12			33.67	1.52E+03	33.55	2.90E+04	N			37.16	1.36E+02	UD	0	N
	#3	3-Jul	#0	0	UD	0	26.72	1.87E+05	28.14	5.32E+05	P			20.08	1.85E+07	20.16	3.90E+07	N
		6-Jul	#3	3			13.6	1.64E+09	14.5	8.19E+08	P			14.59	8.25E+08	15.34	5.21E+08	P
		8-Jul	#5	5			19.64	2.50E+07	20.49	3.26E+07	N			23.54	1.68E+06	24.3	4.20E+06	N
		10-Jul	#7	7			25.92	3.25E+05	27.26	8.55E+05				35.29	4.96E+02	36.73	5.24E+03	N
	#4	18-Jul	#0	12			13.07	2.36E+09	12.66	2.20E+09	N			24.35	9.62E+05	23.37	6.93E+06	N
		20-Jul	#2	14			22.12	4.50E+06	21.39	2.01E+07	N			22.08	4.63E+06	21.86	1.56E+07	N
		22-Jul	#4	16			31.8	5.55E+03	32.73	4.50E+04				UD	0	UD	0	
		24-Jul	#6	18			31.68	6.03E+03	31.26	9.93E+04				26.28	2.53E+05	25.53	2.17E+06	
		27-Jul	#9	21			33.93	1.27E+03	31.76	7.59E+04				27.66	9.73E+04	27.73	6.64E+05	
	#5	28-Aug	#0	1			21.08	9.24E+06	20.8	2.76E+07	P			21.63	6.32E+06	21.18	2.25E+07	P
		31-Aug	#3	4			24.37	9.49E+05	24.18	4.48E+06				22.01	4.86E+06	21.98	1.46E+07	
		3-Sep	#6	7			33.44	1.78E+03	32.32	5.62E+04				20.57	1.32E+07	20.62	3.04E+07	
		7-Sep	#10	11			30.87	1.06E+04	30.35	1.62E+05	N			32.3	3.93E+03	30.99	1.15E+05	N
		10-Sep	#13	14			23.75	1.46E+06	23.69	5.83E+06				23.34	1.93E+06	22.33	1.21E+07	
	#6	27-Dec	#0	0			13.63	1.60E+09	14.15	9.88E+08	P			22.09	4.59E+06	21.42	1.98E+07	N
		28-Dec	#1	1			24.44	9.04E+05	23.88	5.27E+06	P			14.1	1.16E+09	13.42	1.46E+09	P
		31-Dec	#4	4			32.17	4.29E+03	34.63	1.62E+04	N			26.55	2.10E+05	26.16	1.54E+06	N
Lopinavir/ ritonavir	#1	6-Feb	#0	-2	21.97	6.39E+07	21.01	9.70E+06	21.34	2.07E+07	P	27.59	1.89E+06	27.22	1.32E+05	28.11	5.41E+05	P
		7-Feb	#1	-1	UD	0	UD	0	UD	0	N	30.17	3.77E+05	27.75	9.15E+04	28.46	4.48E+05	N
		8-Feb	#2	0	31.64	1.50E+05	31.74	5.78E+03	32.21	5.96E+04	N	31.75	1.40E+05	29.66	2.44E+04	30.07	1.88E+05	N
		9-Feb	#3	1	UD	0	UD	0	UD	0	N	32.22	1.04E+05	32.46	3.51E+03	33.05	3.79E+04	N
		10-Feb	#4	2	UD	0	UD	0	UD	0	N	31.76	1.39E+05	29.88	2.09E+04	30.63	1.39E+05	N
		11-Feb	#5	3	UD	0	NA		NA			33.71	4.11E+04	32.43	3.59E+03	33.46	3.04E+04	N
		12-Feb	#6	4	UD	0	UD	0	UD	0	N	UD		NA		NA		
		13-Feb	#7	5	37.38	4.13E+03	UD	0	UD	0	N	UD		NA		NA		
	#2	2-Mar	#0	7	UD	0	UD	0	UD	0	N	UD	0	UD	0	UD	0	N
		4-Mar	#2	9	UD	0	UD	0	UD	0	N	37.12	4.86E+03	37.27	1.26E+02	37.27	3.92E+03	N
		6-Mar	#4	11			35.29	4.96E+02	35.83	8.50E+03		UD	0	UD	0	UD	0	
		9-Mar	#7	14	UD	0	36.30	2.46E+02	36.26	6.74E+03		UD	0	UD	0	UD	0	
		11-Mar	#9	16	UD	0	UD	0	UD	0								
		16-Mar	#14	21	NA		NA		UD	0	N	27.17	2.46E+06	NA		UD	0	N
	#3	28-Feb	#0	1	32.95	6.61E+04	21.40	7.41E+06	21.87	1.55E+07	P	20.07	2.10E+08	18.03	7.63E+07	18.00	1.25E+08	P
		2/29	#3	4	29.16	7.09E+05	30.17	1.71E+04	30.39	1.59E+05	N	28.64	9.81E+05	33.35	1.90E+03	34.75	1.52E+04	N
		4-Mar	#5	6	33.84	3.79E+04	34.48	8.68E+02	36.50	5.93E+03	N	29.22	6.83E+05	34.60	7.99E+02	33.88	2.43E+04	N
		9-Mar	#10	11	34.94	1.90E+04	32.52	3.37E+03	34.07	2.19E+04		30.83	2.49E+05	UD	0	UD	0	
		11-Mar	#12	13	26.36	4.09E+06	26.85	1.71E+05	27.42	7.84E+05		29.18	7.00E+05	35.63	3.92E+02	35.15	1.23E+04	
		13-Mar	#14	15	32.30	9.93E+04	37.43	1.13E+02	37.27	3.92E+03		36.04	9.55E+03	33.23	2.06E+03	34.36	1.87E+04	
		16-Mar	#17	18	37.04	5.11E+03	36.50	2.15E+02	36.22	6.89E+03		32.56	8.44E+04	33.28	1.99E+03	31.34	9.52E+04	
		18-Mar	#19	20	UD	0	36.08	2.87E+02	35.35	1.10E+04		UD	0	UD	0	UD	0	
		20-Mar	#21	22	UD	0	36.42	2.27E+02	36.25	6.78E+03		NA		UD	0	UD	0	
	#4	10-Mar	#0	9	24.76	1.11E+07	25.47	4.43E+05	25.30	2.45E+06	P	NA		27.25	1.29E+05	25.57	2.12E+06	
		13-Mar	#3	12	UD	0	35.02	5.98E+02	35.20	1.19E+04	N	25.34	7.74E+06	35.32	4.86E+02	36.08	7.43E+03	N
		16-Mar	#6	15	NA		34.60	7.99E+02	35.38	1.08E+04		UD	0	UD	0	UD	0	
		18-Mar	#8	17	UD	0	35.30	4.92E+02	36.07	7.47E+03		UD	0	30.65	1.23E+04	29.82	2.16E+05	
		20-Mar	#10	19	UD	0	32.19	4.24E+03	32.84	4.25E+04		UD	0	UD	0	UD	0	
		23-Mar	#13	22	UD	0	39.22	3.27E+01	UD	0	N	NA		UD	0	UD	0	N

* Pat. no, Patient number; UD, undetermined; NA, not assayed; Ct, cycle threshold; Day 1, Hospitalization day; Day 2, days after symptom onset; E, envelope protein; RdRp, RNA dependent RNA polymerase; P, Positive; N, Negative;
