# Supplementary figures and images for: Comparable Efficacy of Lopinavir/Ritonavir and Remdesivir in Reducing Viral Load and Shedding Duration in Patients with COVID-19
Source: Microorganisms. 2024 Aug 16;12(8):1696. doi: 10.3390/microorganisms12081696 (PMC11357406; doi:10.3390/microorganisms12081696)

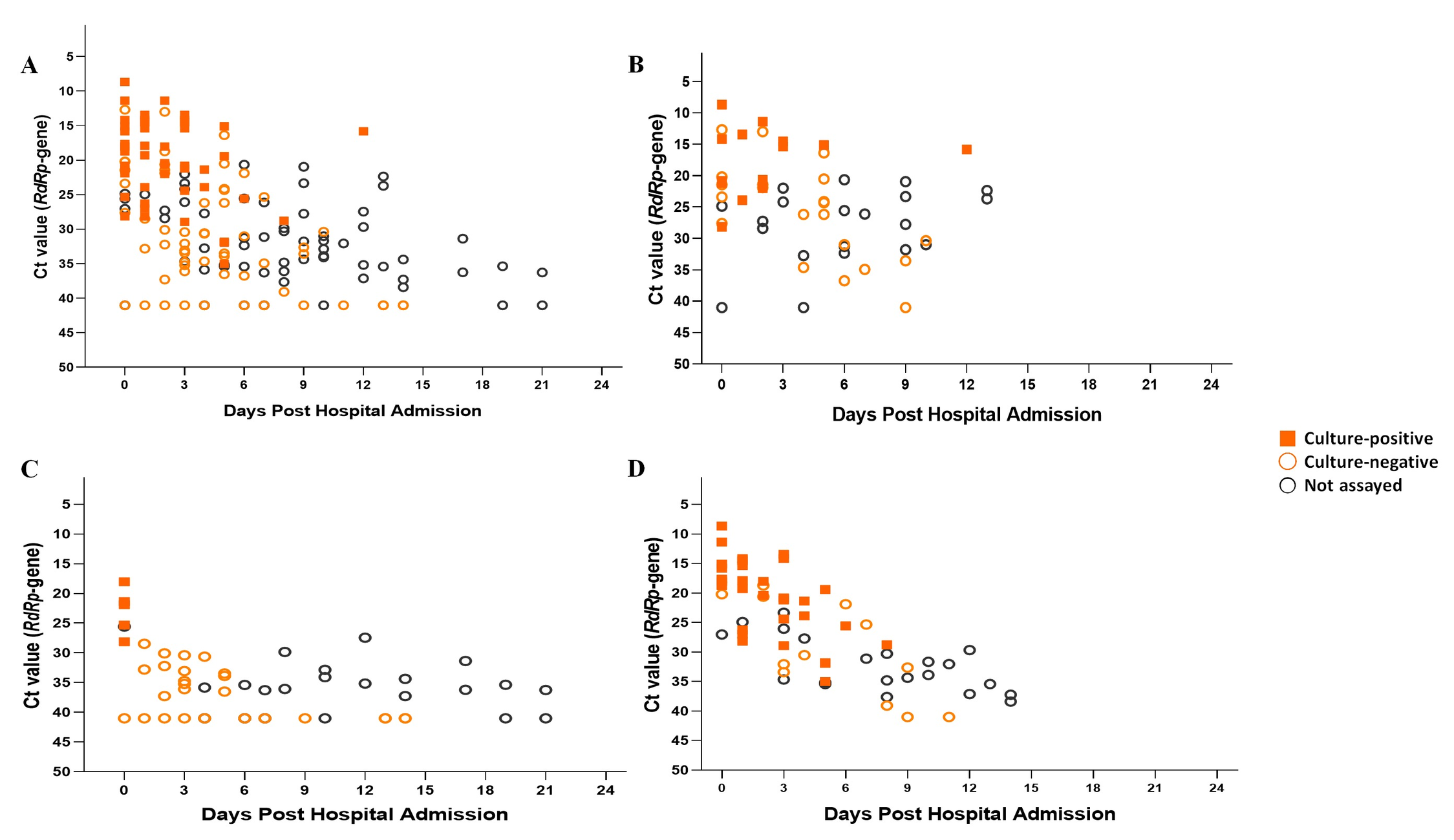

Supplement: Supplementary file 1 [file microorganisms-12-01696-s001.zip › Figure S1_MDPI.tif]

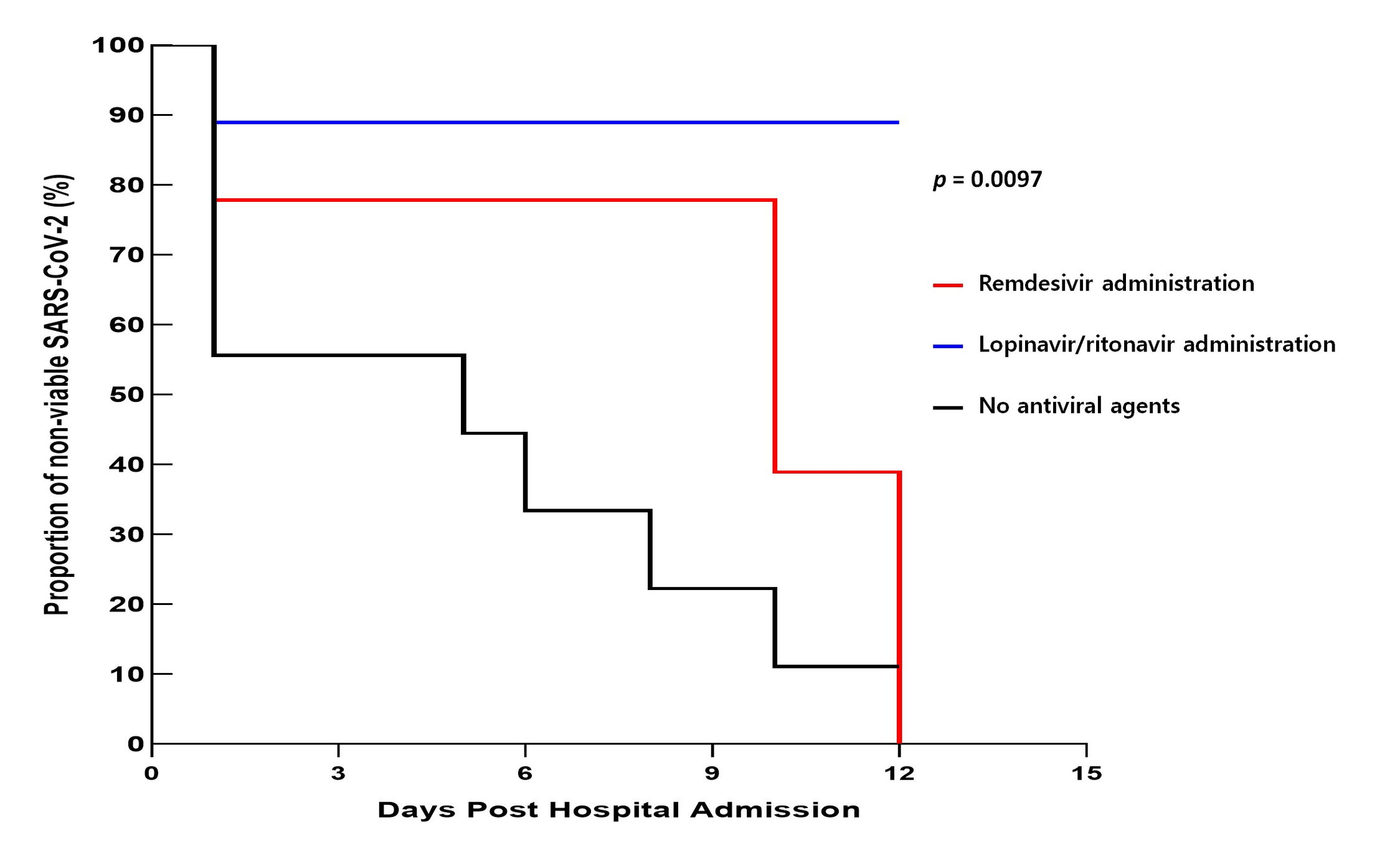

Supplement: Supplementary file 1 [file microorganisms-12-01696-s001.zip › Figure S2_MDPI.tif]

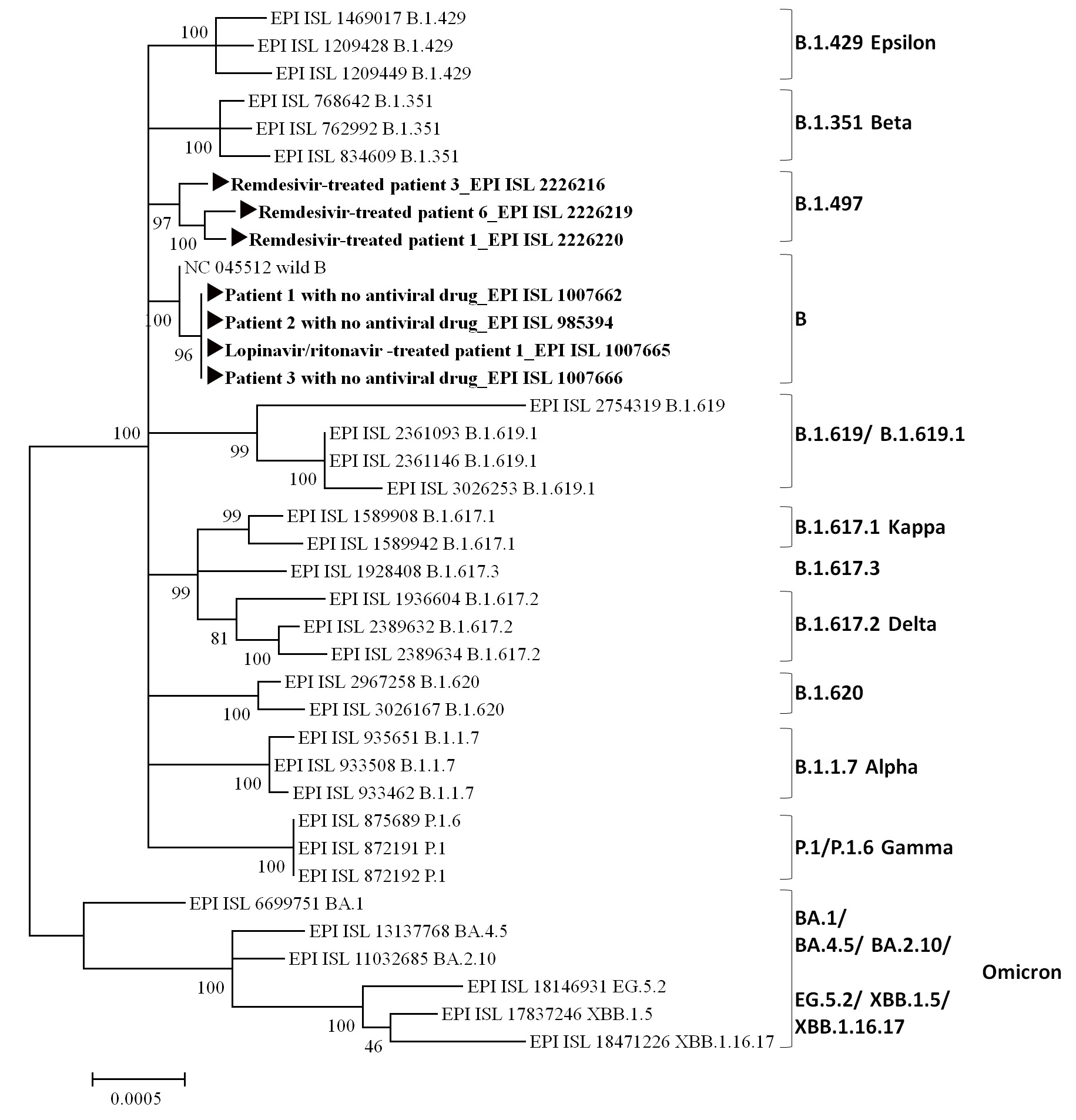

Supplement: Supplementary file 1 [file microorganisms-12-01696-s001.zip › Figure S3_MDPI.tif]
